# Skull Sex Estimation Based on Wavelet Transform and Fourier Transform

**DOI:** 10.1155/2020/8608209

**Published:** 2020-01-11

**Authors:** Wen Yang, Mingquan Zhou, Pengfei Zhang, Guohua Geng, Xiaoning Liu, Haibo Zhang

**Affiliations:** ^1^College of Information Science and Technology, Northwest University, Xi'an, China; ^2^College of Information Science and Technology, Beijing Normal University, Beijing, China

## Abstract

Skull sex estimation is one of the hot research topics in forensic anthropology, and has important research value in the fields of criminal investigation, archeology, anthropology, and so on. Sex estimation of skull is crucial in forensic investigations, whether in legal situations that involve living people or to identify mortal remains. The aim of this study is to establish a skull-based sex estimation model in Chinese population, providing a scientific reference for the practical application of forensic medicine and anthropology. We take the superior orbital margin and frontal bone of the skull as the research object and proposed a technology of objective sex estimation of the skull using wavelet transform and Fourier transform. Firstly, the supraorbital margin and frontal bone were quantified by wavelet transform and Fourier transform, and then the extracted features were classified by SVM, and the model was tested. The experimental results show that the accuracy rate of male and female sex discrimination is 90.9% and 94.4%, respectively, which is higher than that of morphological and measurement methods. Compared with the traditional methods, the method has more theoretical basis and objectivity, and the correct rate is higher.

## 1. Introduction

In the practice of forensic identification, it is often the case that a corpse is dismembered and highly decomposed leaving only skeletal remains, or that only skeletal remains are left in a major disaster. It is very important to get the identity information of the dead from the fragmentary bones. Sex estimation is the first and key step of skeletal remains identification, which can reduce the number of possible identity matching by nearly 50%, and also provide basic reference for face reconstruction. Human identity is not only a prerequisite for an individual who officially declares death but also a basis for tracing people, investigating crime, mass disasters, or war atrocities [1, 2].

The identification of unknown skeletal remains is an important part of anthropological and forensic research. Sex estimation of unknown bones is a very important part of anthropological and forensic analysis. In the context of forensic analysis, sex estimation together with assessment of racism, population affinity, and stature is important for the process of individual identification. The sex estimation based on skeletal remains is based on sexual dimorphism, which is generally present to varying degrees in bones of human skeleton. Pelvis and skull are the most widely used sites for determining skeletal sex [3, 4]. However, the pelvis is not easy to store and fragile, and the skull is composed of hard tissue, which in most cases can be well preserved, and the stability of the sexual dimorphic features is better [5, 6]. Therefore, the skull has become the most commonly used bones in sex estimation, and skull sex estimation has important research significance and application value.

The traditional methods of skull sex estimation are mainly morphological discrimination and measurement discrimination. Morphological discrimination mainly relies on the expert's understanding of the differences in morphological characteristics between male and female skulls. Experts' subjective understanding of skull morphological features has an important influence on sex estimation. The measurement discriminant method refers to first identifying the feature points of the skull, measuring some sex-specific feature index items of the skull, and then using these geometric quantities to establish a discriminant function to determine the sex.

In the morphological approach, anthropologists compare visual forms of unknown skulls and draw conclusions through visual observation and experience. Wells [7] used the anthropologists and standard methods in the field of skull sex to assess the sex of the skull. Studies have shown that cognitive bias has an impact on the sex estimation of the skull, and the skull is effective in identifying sex in human skeletal remains. Krogman [8] used morphological methods to identify 750 skulls of known sex. The correct rate was 82–87%. Ramsthaler et al. [9] used kappa statistics to quantify the differences between two different observers in sex visual morphology assessment, and the consistency was only 90.8%. Therefore, the visual form assessment method is subjective, low in reliability, low in recognition rate, and theoretically insufficient.

The measurement discriminant method is further divided into physical measurement methods and computer-aided measurement methods. The physical measurement method is to measure the physical object of the skull and establish a sex discriminant function. Some scholars have done a lot of research using this method and have achieved good results [10–14]. However, the biggest disadvantage of this method is that it may cause secondary damage to the skull during the measurement process, and the measurement results are not accurate enough due to human factors. With the rapid development of computer technology, computer-aided measurement is increasingly used for the measurement of skull feature items. This measurement method is to digitalize the skull object, generate the three-dimensional model of the skull, and realize the measurement with the help of computer program or software. At present, the method of computer-aided measurement is the most commonly used method, and many scholars [15–23] use this method to study the sex problem of the skull. Compared with the morphological method, this method is more objective, but the measurement accuracy is still difficult to guarantee. Williams and Rogers [24] showed that when different observers measured some sex differences, most of the measurement variables had errors of more than 10%.

**Figure 1:**
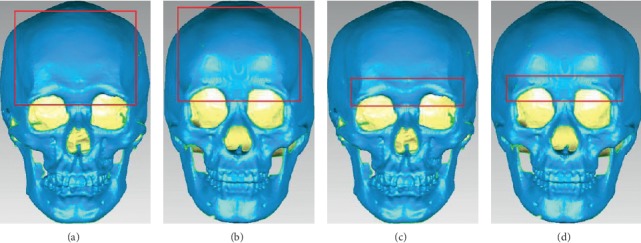
Difference of frontal bone and supraorbital margin between male and female skulls. (a) Frontal bone of male skull. (b) Frontal bone of female skull. (c) Supraorbital margin of male skull. (d) Supraorbital margin of female skull.

The morphologies of the frontal bone and superior orbital margin are commonly used skull features with sexual dimorphism [23, 25–28]. The male cranial forehead is more inclined, the frontal nodules are not obvious, and the nasal root sag is deeper, while the female cranial forehead is steeper, the frontal node is large and obvious, and the nasal root depression is shallow [23]. Male skulls are characterized by a wider, rounded, “blunt thick” supraorbital margin, while female skulls typically have a thin “sharp, like the edge of a slightly dulled knife” supraorbital margin [29], as shown in Figure 1.

In traditional methods, the accuracy is very low when these two commonly used features are used as skull sex estimation features. Studies have shown that even if two experienced experts use the visual observation method to estimate the sex of the skull, at least 50% of the observations differ [30]. For these reasons, alternative approaches have been developed based on premises other than simple visual assessment and measurement methods. One of these approaches is the introduction of shape analysis into forensic anthropological, namely, thin-plate splines [31] and Euclidean distance matrix analysis [32]. Alternatively, Schiwy-Bochat [33] attempted to quantify the roughness of the supranasal (glabellar) region for sex estimation from photographs by applying the box-counting method from fractal geometrics. Although advances have been made in 3D laser or CT scanning technology, most of the studies use landmark-based or contour-based approaches rather than surface quantification.

Based on the above analysis, we propose a new method for skull sex estimation. In the sex estimation, we first use wavelet analysis to quantify the supraorbital margins of the skull and quantify the frontal sagittal arc using Fourier transformation. Then, we fuse the quantified features and construct a sex classifier using support vector machine method, which ultimately achieves the sex identification of the skull.

Compared with the traditional methods, the innovation and the main contribution of this method are: (1) it gives a clear explanation about the importance of the skull local area for classification, (2) it proposes a promising tool for inexperienced observers to determine the sex of a skull without much human-computer interaction, and (3) it is without tedious manual measurement, quantifies the features, the results are more objective and accurate, and got rid of the influence of the skull size.

**Figure 2:**
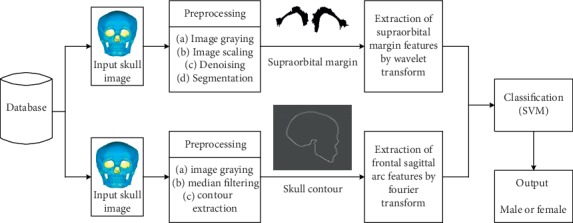
Method flow chart of this paper.

The method flow chart of this paper is shown in Figure 2.

## 2. Materials

The skull data used in this study are from Han Adults in northern China. Under the principle of informed consent, the CT images of living human head were obtained by using the multirow detector spiral CT scanner made by Siemens in Germany. The three-dimensional modeling of the skull was realized by computer software developed by ourselves, and the three-dimensional model data of the skull were obtained and saved in OBJ format. A total of 133 skulls, 73 males and 60 females, aged between 22 and 28, were collected. All skulls are essentially intact; that is, each skull contains all the bones from the parietal bone to the jaw bone and has intact teeth. In addition, their birth date, sex, and residence information were recorded in detail. Patients who had undergone craniofacial surgery, cleft lip or palate or other craniofacial lesions or with syndrome were excluded. This study has been approved by Northwest University ethics committee. Distribution of skulls in male and female of all ages is shown in Table 1.

## 3. Methods

### 3.1. Quantitative Extraction of Supraorbital Margin Features

#### 3.1.1. Segmentation of Supraorbital Margins

The segmentation process of supraorbital margins mainly includes the following steps:

1. Skull image acquisition: the image used here is a skull image that is completely separated from the skeleton. The skull image data are used in this paper to import the three-dimensional model of the skull into the Geomagic Studio 12.0 software and obtain the frontal image of the skull through the image acquisition function of the software. The supraorbital margins region of all images is complete and clear.
2. Image gray processing: the skull image obtained in the previous step is a color image, and the color image should be further converted into a gray image. This is because color information has no effect on sex and reduces the amount of calculation.
3. Image scaling: to ensure accuracy, we scale the image to increase the image pixel rate.
4. Noise reduction: in the denoising process, the median filter is used to smooth the image, protect the edge information, and increase the image clarity. The main reason to increase the clarity of the image will be clearly able to identify the shape of the supraorbital region for sex estimation of the skull.
5. Segmentation: it is the process of extraction of required part of the image. Here, it is necessary to clearly extract the supraorbital margin region to determine the sex of the skull. In this paper, the fuzzy C-means clustering algorithm is used to realize the segmentation process.

#### 3.1.2. Quantitative Extraction of Supraorbital Margins

Let **x**(*x*, *y*) ∈ **R**^2^ be the vectorial notation of the 2D point. The continuous wavelet transform (CWT) of a 2D signal *f*(*x*) is defined as follows [34]:

(1) $$\begin{array}{c} W_{\psi }\left(b,a\right)=\frac{1}{\sqrt{a}}\int \psi^{*}\left(\frac{x-b}{a}\right)f\left(x\right)\text{d}x, \end{array}$$

where *ψ*^*∗*^, **b**, and *a* represent the complex conjugate of the analyzed wavelet *ψ* (or “mother wavelet”), the shifting parameter, and the dilation parameter (related to analyzed scale), respectively.

Depending on the type of information extracted from the signal, choose to use a different mother wavelet. For example, Morlet wavelets are suitable for local frequency analysis, while Mexican hats are suitable for edge detection in image processing. Differential wavelets, such as the derivatives of the Gaussian, are particularly suitable for analyzing singularities and extracting differential information from the signal. Therefore, in this work, we used the first partial derivatives with respect to *x* and *y* of the 2D Gaussian function *ϕ*(*x*, *y*) denoted as *ψ*_*x*_ and *ψ*_*y*_:

(2) $$\begin{array}{c} \psi_{x}\left(x,y\right)=\frac{\partial \phi \left(x,y\right)}{\partial x},\\ \psi_{y}\left(x,y\right)=\frac{\partial \phi \left(x,y\right)}{\partial y}, \end{array}$$

where the 2D Gaussian function is

(3) $$\begin{array}{c} \phi \left(x,y\right)=\exp\left[\frac{-\left(x^{2}+y^{2}\right)}{2}\right]=\exp\left[\frac{-\left({\left|x\right|}^{2}\right)}{2}\right]. \end{array}$$

In order to study morphological variation of supraorbital margin, we have assessed the potential of the wavelet for numerical multiscale estimation of the gradient field of morphological shapes. Therefore, we have applied the 2D wavelet transform (*W*_*ψ*_*x*__ and *W*_*ψ*_*y*__) in the expanded surface for the purpose of calculating the gradient ∇*W*, i.e.,

(4) $$\begin{array}{c} \nabla W=\left(W_{\psi_{x}},W_{\psi_{y}}\right)=\left(\frac{\partial f\left(x,y\right)}{\partial x},\frac{\partial f\left(x,y\right)}{\partial y}\right). \end{array}$$

The gradient-based measurement method has the advantages of translation and rotation invariance, which can ensure that the anatomical correspondence of the supraorbital margin does not depend on the angle and direction of the scanner. The entropy can be measured from the gradient field to achieve a quantitative analysis of the disorder degree of the surface vector direction, i.e., the entropy of the orientation distribution *E*_∇*W*_, denoted as

(5) $$\begin{array}{c} E_{\nabla W}=-\sum \rho \left(\theta \right)\log\;\rho \left(\theta \right), \end{array}$$

where *ρ*(*θ*) is the probability of angle *θ* being incremented.

**Figure 3:**
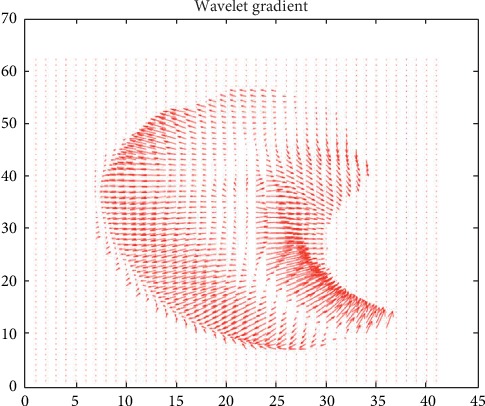
Gradient field of the points (voxels) belonging to the original supraorbital surface.

The entropy is maximized when the probability of each measurement is the same; thus, a high entropy value means that the angles of the gradient field are evenly distributed. As discussed earlier, the region of morphologic importance is the valley region of the supraorbital margin, where the high variation of the arrangement of the gradient vectors compared with other regions can be seen in Figure 3.

Considering a small neighborhood of voxels at each point (*x*, *y*) on the surface, the entropy is calculated locally, and its value is assigned to the texture value of the supraorbital margin surface. Features are extracted from the surface texture of entropy to describe the degree of sex dimorphism in the supraorbital margin. Further, the valley region of the supraorbital margin is divided by the method based on threshold, which is convenient for subsequent measurements. There are two main thresholds here: one for the surface height value *th* and another for the entropy *te*. Two measures were obtained from the segmentation: area and thickness, which was defined as the number of erosions necessary to completely erode the area [35].

### 3.2. Quantitative Extraction of Frontal Bone Morphological Features

After obtaining the skull image using Geomagic Studio 12.0 software, the image is grayscaled and median filtered, and then the processed image is extracted by canny contour to obtain the lateral contour of the skull.

Frontal bones are important regions of skull sex differences. Here, we use Fourier transform to quantify the data of these two nonmeasurable features. Firstly, 18 points were calibrated in the frontal bone regions of skull profile, and the frontal line of three-dimensional skull was fitted by the cftool curve fitting toolbox of MATLAB. Secondly, the spatial curve is optimized by Levenberg–Marquardt algorithm. Finally, the three-dimensional space curve is projected to the two-dimensional plane *XY*, and the projection curve *S* is Fourier transformed.

Fourier transform is used to quantify the shape of the curve, i.e., divide the 32 segments on the *X* axis and then calculate the *Y* value of the corresponding points on the curve. Apply formula (6) to obtain *A*_0_, apply formulas (7) and (8) to calculate 16 cosine coefficients *A*_*k*_ and 16 sine coefficients *B*_*k*_, respectively, and then synthesize into 16 amplitudes *P*_*k*_ using formula (9); finally, *P*_*k*_ is normalized to *P*_*k*_′ using formula (10).

(6) $$\begin{array}{c} A_{0}=\frac{1}{32}\overset{31}{\underset{m=0}{\sum }}Y_{m}, \end{array}$$

(7) $$\begin{array}{c} A_{k}=\frac{1}{16}\overset{31}{\underset{m=0}{\sum }}Y_{m}\times \;\cos\left(2\cdot \pi \cdot k\cdot \frac{m}{32}\right), \end{array}$$

(8) $$\begin{array}{c} B_{k}=\frac{1}{16}\overset{31}{\underset{m=0}{\sum }}Y_{m}\times \;\sin\left(2\cdot \pi \cdot k\cdot \frac{m}{32}\right), \end{array}$$

(9) $$\begin{array}{c} P_{k}=\sqrt{A_{k}^{2}+B_{k}^{2}}, \end{array}$$

(10) $$\begin{array}{c} P_{k}^{\prime }=P\times \frac{100}{A_{0}}\;\left(k=1,2,\ldots ,16\right). \end{array}$$

### 3.3. Classification Method

Support vector machine (SVM) is a learning algorithm for pattern classification and regression. The basic training principle of SVM is to find the optimal linear hyperplane, which minimizes the expected classification error of unknown test samples, that is, good generalization performance. Because SVM has good learning ability and can solve the problems of small sample, nonlinearity, and high-dimensional classification, it has become the preferred classifier to deal with sex estimation.

#### 3.3.1. Support Vector Classifier

Given a labeled set of *M* training samples (*x*_*i*_, *y*_*i*_), where *x*_*i*_ ∈ *R*^*N*^ and *y*_*i*_ ∈ *R*^*N*^*y*_*i*_ ∈ {−1,1} are associated. SVM classifier finds a small part of the data points of the correct maximum separation hyperplane and maximizes the distance from any class to the hyperplane. Vapnik [36] showed that maximizing the margin is equivalent to minimizing the VC dimension when constructing the optimal hyperplane. Computing the optimal hyperplane is a constrained optimization problem that can be solved using quadratic programming techniques. The discriminant hyperplane is defined by the level set as follows:

(11) $$\begin{array}{c} f\left(x\right)=\overset{M}{\underset{i=1}{\sum }}y_{i}\alpha_{i}\cdot k\left(x,x_{i}\right)+b, \end{array}$$

where *k*(·, ·) is a kernel function, and the sign of *f*(*x*) determines the membership of *x*. Constructing an optimal hyperplane is equivalent to finding all nonzero values *α*_*i*_. Any vector *x*_*i*_ corresponding to nonzero *α*_*i*_ is the support vector of the optimal hyperplane.

For linear SVM, the kernel function is only a simple point product in the input space, while the kernel function in nonlinear SVM effectively projects the sample to a higher (possibly infinite) dimension feature space through the nonlinear mapping function: Φ : *R*^*N*^⟶*F*^*N*^, *M* ≫ *N*. Then, construct a hyperplane in *F*. The motive behind this kind of mapping is that it is more likely to find linear hyperplane in high-dimensional feature space. Using Mercer's theorem [37], the samples are projected into the high-dimensional feature space, which can be calculated by the following formula:

(12) $$\begin{array}{c} k\left(x,x_{i}\right)=\Phi \left(x\right)\cdot \Phi \left(x_{i}\right), \end{array}$$

where Φ(*x*) is the mapping function of projection from low-dimensional space to high-dimensional space and · is the inner product operation.

In order to maximize the separation hyperplane interval 2/‖*w*‖ and minimize the error ∑_*i*=1_^*l*^*ξ*_*i*_ between training samples, the penalty parameter *C* is introduced [38]. The convex quadratic programming problem can be expressed as follows:

(13) $$\begin{array}{c} \min_{\gamma ,\omega ,b}\frac{1}{2}{\left\|\omega \right\|}^{2}+C\overset{m}{\underset{i=1}{\sum }}\xi_{i},\xi_{i}\geq 0,\;i=1,\ldots ,m,\\ y^{\left(i\right)}\left(\omega^{T}x^{\left(i\right)}+b\right)\geq 1-\xi_{i},\;i=1,\ldots ,m, \end{array}$$

where *C* is a constant and *C* > 0. When *C* is larger, it means that the punishment for sex judgment error is larger; when *C* is smaller, the punishment for sex judgment error is smaller.

In order to obtain the best separating hyperplane in quadratic programming, a Lagrangian operator is constructed to realize the solution, and the following formula is obtained:

(14) $$\begin{array}{c} \Gamma \left(\omega ,b,\xi ,\alpha ,r\right)=\frac{1}{2}\omega^{T}\omega +C\overset{m}{\underset{i=1}{\sum }}\xi_{i}-\overset{m}{\underset{i=1}{\sum }}\alpha \left[y^{\left(i\right)}\left(x^{T}\omega +b\right)-1+\xi_{i}\right]-\overset{m}{\underset{i=1}{\sum }}r_{i}\xi_{i}, \end{array}$$

where *α*_*i*_ and *r*_*i*_ are Lagrange multipliers.

Taking formula (14) as a function of variables *ω* and *b*, the partial derivatives of them are obtained, and the expressions of *ω* and *b* are obtained. Then, substitute it into formula (14) to find its maximum value. Finally, the following formula is obtained:

(15) $$\begin{array}{c} \max_{\alpha }W\left(\alpha \right)=\overset{m}{\underset{i=1}{\sum }}\alpha_{i}-\frac{1}{2}\overset{m}{\underset{j=1}{\sum }}y^{\left(i\right)}y^{\left(j\right)}\alpha_{i}\alpha_{j}<x^{\left(i\right)},x^{\left(j\right)}>0\leq \alpha_{i}\leq C,\;i=1,2,\ldots ,m,\\ \overset{m}{\underset{i=1}{\sum }}\alpha_{i}y^{\left(i\right)}=0, \end{array}$$

where *α*_1_, *α*_2_,…, *α*_*m*_ needs to satisfy the conditions of semipositive definite and nonnegative constraints.

#### 3.3.2. Kernel Function and Optimal Parameter Selection

The accuracy of sex estimation is directly affected by kernel function. After comparing and analyzing all kinds of kernel functions of SVM, radial basis function (RBF) is selected as the kernel function of skull feature mapping. RBF can fit the continuous function on the skull data set as accurately as possible [39], and its mathematical expression can be expressed as follows:

(16) $$\begin{array}{c} k\left(\left\|x-x_{i}\right\|\right)=\exp\left\{\frac{{\left(\left\|x-x_{i}\right\|\right)}^{2}}{\delta^{2}}\right\}, \end{array}$$

where *x*_*i*_ is the center of kernel function and *δ* is the width of kernel function, which controls the radial action range of kernel function.

In the training stage of sex estimation, parameters *C* and *δ* have the greatest influence on the effect of sex estimation. The change of parameter *C* can significantly separate the samples with correct classification from those with wrong classification. When *C* is larger, the classification error rate is smaller, but the interval is smaller; when *C* is smaller, the interval is larger, but the classification error rate is larger. The change of parameter *δ* directly affects the calculation ability of kernel function, thus further affecting the effect of sex estimation. When the *δ* is larger, there may be misjudgment, that is, the training samples or test samples are divided into the same category; when the *δ* is smaller, it is easy to have over fitting phenomenon, that is, it can correctly classify the sex of the training skull samples, but the classification accuracy of the test skull samples is not high, and the generalization ability is poor. Therefore, it is very important to select the appropriate parameters *C* and *δ* for sex estimation.

The common methods to optimize the parameters *C* and *δ* are grid search, genetic algorithm, and chaos optimization algorithm. In this paper, the algorithm in [40] is used to determine the appropriate parameters *C* and *δ*. Set the range of the parameters *C* and *δ*, 2^−5^ ≤ *C* ≤ 2^15^, 2^−15^ ≤ *δ* ≤ 2^5^, and the step size is set to 0.5 to obtain *C* values and *δ* values. The SVM model is used to classify the skull samples and obtain the sex estimation accuracy rate. The optimal parameters *C* and *δ* are determined according to the sex estimation accuracy rate.

Firstly, the quantified supraorbital margin features, frontal bone morphological features, are fused to form the optimal feature set. Then, the SVM classification model is trained according to the optimal feature set and tested. Finally, sex estimation of the unknown skull is realized.

## 4. Experiment and Results

### 4.1. Fitting Results of Frontal Bone Morphology

**Figure 4:**
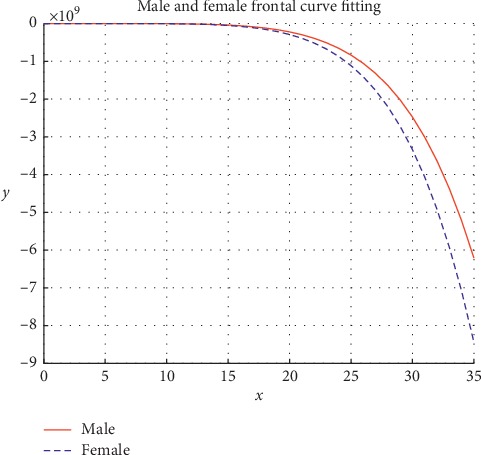
Frontal line diagram.

We used the method described in Section 3.2 to fit the frontal bone curve, and then projected the frontal bone curve of male and female to the *XY* plane. The frontal bone line after projection was obtained as shown in Figure 4.

Using the cftool curve fitting toolbox of MATLAB, the frontal bone lines of male and female were fitted. The fitted curve equations of men and women were as follows:

*y*_1_=−8.6663 − 1.4380 · *x* − 2.3911 · *x*^2^ − 3.9862 · *x*^3^+1.0611 · *x*^4^ − 4.0991 · *x*^5^ − 3.2628 · *x*^6^   and*y*_2_=−16.3129 − 5.0763 · *x* − 7.2886 · *x*^2^+0.1792 · *x*^3^ − 0.0003 · *x*^4^+2.0856 · *x*^5^ − 4.6519 · *x*^6^ The Fourier transform method is used to quantify the shape of frontal bone lines of male and female. The axes of two-dimensional curves are divided into 32 parts, and the corresponding values on the curves are calculated. Finally, the synthetic amplitude is calculated as the measurement index of sex estimation. Fourier transforms were used for frontal sagittal arc morphology, and a total of 16 sex estimation measurements were obtained.

### 4.2. Quantitative Results of Supraorbital Margin

We randomly selected 23 skull samples from 133 skulls for testing. Among them, there are 13 males and 10 females. The results are based on measurements of the supraorbital margin valley. The experimental environment is as follows: implemented using Matlab R2015a, all the experimental programs are executed on personal computers with 3.40 GHz CPU and 8G RAM. Four different values are used for the threshold *th*, varying from 1 to 4 voxels, which are selected based on observations of the entropy surface. For the threshold *te*, we used four empirically chosen values: 0.5, 0.6, 0.7, and 0.8. The features were obtained for five different scales of the 2D wavelet.

**Figure 5:**
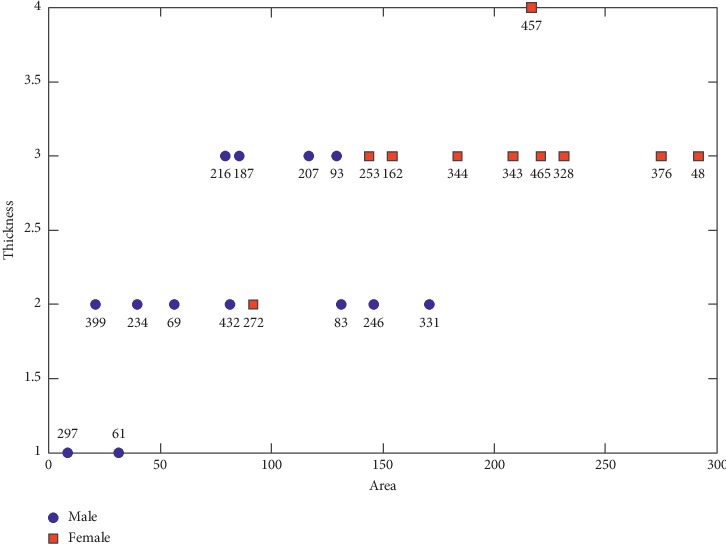
Two-dimensional feature space of area and thickness of valley region considering scale is 0.00005, *th* = 2 and *te* = 0.8.

Figure 5 presents the two-dimensional feature space defined by the area and thickness of the valley region with respect to the two classes of individuals: male and female. In all parameter combinations, when *th* = 2, *te* = 0.8, and the scale of the wavelet is 0.00005, this combination has the highest discriminating power. Under the optimal parameters, these features are most effective in distinguishing between male and female skulls.

### 4.3. Classification Results

**Figure 6:**
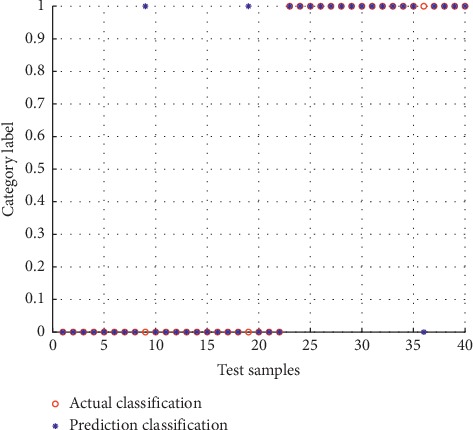
Prediction results of test samples. Label category 0 denotes male and label category 1 denotes female.

In the sex estimation experiment, we divided the experimental data into two parts, 70% as training set and 30% as test set. The SVM method has two important parameters, the penalty parameter C and the parameter A related to the computing power of the kernel function, which have the greatest impact on the results of sex estimation. In this paper, the grid search algorithm is used to solve these two parameters. The range of parameters *C* and *δ* was set to 2^−5^ ≤ *C* ≤ 2^15^, 2^−15^ ≤ *δ* ≤ 2^5^, and the step size was set to 0.5. The optimal parameters *C* = 1.5824 and *δ* = 0.5 for 93 training samples (51 males and 40 females) can be obtained by multiple calculations. After the establishment of the sex estimation model, we tested 40 test samples (22 males and 18 females) and compared them with the records in the database. The results were as follows: among 22 male skulls, 2 were misjudged, 20 were correctly judged, and the correct rate was 90.9%; among 18 female skulls, 1 was misjudged, 17 were correctly classified, and the correct rate was 94.4%. The prediction results of the classification model for the test sample are shown in Figure 6.

## 5. Discussion

In forensic medicine and anthropology, researchers use human bones to identify human remains at every stage [41]. The sex of the remains is one of the most important parts of the identification process. Many new methods and technologies have been used in this research, and traditional methods have been constantly improved and optimized. The pelvis and skull are the most commonly used bones in the study of estimating sex by observing and measuring bones [42]. In this study, the measurement of the supraorbital margin of the skull and the morphology of the frontal was used as an indicator of sex. Different from the traditional measurement method, we quantify the indicator by establishing a mathematical model to obtain the measurement result, which overcomes the error of the measurement process.

In the sex estimation problem, when the characteristics of the supraorbital margin and frontal sagittal arcs are used for the study of cranial dimorphism, the measurement results obtained by mathematical modeling are more objective. Since the mathematical model fits the shape, iteratively optimizes and adjusts the parameters to make them closer to the true shape. In the quantification of the sagittal arc feature of the frontal bone, in order to make the Fourier series more completely show the shape of the frontal sagittal arc, the subdivision is divided as much as possible, so that the curve formed by the inverse transformation is closer to the original shape. Of course, the finer the division, the more complicated the calculation process. In this study, the frontal sagittal arc was divided into 32 equal parts, and the original curve was reproduced better by inverse transformation, which proved that the degree of division is appropriate. When quantifying the features of the supraorbital margin, the combination of quantifying the supraorbital margin into area and thickness has obvious difference between the two sexes. The results show that the values of female are relatively consistent, and the differences between male are relatively large. This is a common characteristic of sexual dimorphism in the human cranial trait [43]. Bogin's research [44] shows that there is a phenomenon of “prolonged maturation” in the growth of male skeleton. The time and extent of growth are highly variable. Therefore, the range of male supraorbital margins varies widely, and the range of female changes is relatively small.

After quantifying the characteristics of the supraorbital margin and the sagittal arc of the frontal bone, the SVM method was used to establish a sex estimation model, and 40 skulls were tested and achieved good results. In order to verify the superiority of the method, we compare it with the morphological method [8] and the measurement method [21], respectively. The correctness rate of this method is the highest, and the accuracy of the first two methods is less than 90%. The correct rate is over 90%. This is because this study avoids the influence of subjective factors and measurement errors in feature quantification, and the features are more accurate. Therefore, the correct rate is also higher. Liu et al. [45] took the frontal bone as the experimental object and used the forward stepwise regression method based on the maximum likelihood estimation to establish the frontal bone sex discrimination model. The accuracy rate of male discrimination was 89.4%, female discrimination was 85.0%, and the average accuracy rate of discrimination was 87.2%. Yang et al. [46] used Fisher's method and logistic regression method to establish sex discriminant equation for skull with frontal bone only, and the discriminant accuracy was 67.9% and 68.7%, respectively. [45, 46] and this paper use the same anatomical region, but the accuracy of this method is significantly higher than them.

Although the method in this paper has achieved good accuracy in sex estimation, in practical application, the higher the accuracy of sex estimation method, the better the reliability of estimation results. There are still some gaps between the accuracy and ideal value of this paper, which is also the power and direction of future research. In addition, the characteristic areas used for sex estimation in this paper are mainly concentrated in the upper half of the skull. Some researchers have proposed that other areas on the skull can also be used for sex estimation, such as the mandible area [17, 47, 48] and the occipital area [16, 49]. Therefore, it is the next research direction to quantify other regions with mathematical methods or machine learning methods and realize skull sex estimation. Although the accuracy rate varies from different researchers, different races, different sample sizes, and different methods, it is of reference value in the application.

## 6. Conclusions

In this study, we took the skulls of Han adults in northern China as the research object, and realized the sex estimation based on skulls. We use the supraorbital margin and frontal sagittal arc of skull as characteristic indicators and use SVM to establish a sex estimation model to achieve sex identification. Firstly, the feature indexes are quantified, the feature of the supraorbital margin is quantified by two-dimensional wavelet transform, and the feature of frontal sagittal arc is quantified by Fourier transform. Then, the quantized features are fused, and the classification model is trained by the SVM method according to the optimal feature set. Finally, the model is tested to verify its performance.

The advantages of this research work are as follows: firstly, it needs no professional qualification; secondly, when quantified, it can fully approximate the true shape of the skull; and finally, it can get a high recognition rate. Although we use CT scanning to construct the 3D point cloud model of skull, this method can also be used to construct the 3D model in any other way, such as laser scanning and 3D camera. Next, we should collect a larger sample bank to build a model of sex estimation, which will be used in forensic and anthropological fields for the practical application of unknown skeletal sex estimation.

## Figures and Tables

**Table 1:** Distribution of skulls in males and females of all ages.

Age (years)	Male	Female	Total
22	10	7	17
23	12	10	22
24	14	12	26
25	12	10	22
26	10	9	19
27	8	6	14
28	7	6	13
Total	73	60	133

## Data Availability

The .obj format 3D model data used to support the findings of this study may be released upon application to the Northwest University Visual Technology Institute via ghgeng@nwu.edu.cn.
